# Examination of the predicted prevalence of Gitelman syndrome by ethnicity based on genome databases

**DOI:** 10.1038/s41598-021-95521-6

**Published:** 2021-08-09

**Authors:** Atsushi Kondo, China Nagano, Shinya Ishiko, Takashi Omori, Yuya Aoto, Rini Rossanti, Nana Sakakibara, Tomoko Horinouchi, Tomohiko Yamamura, Sadayuki Nagai, Eri Okada, Yuko Shima, Koichi Nakanishi, Takeshi Ninchoji, Hiroshi Kaito, Hiroki Takeda, Hiroaki Nagase, Naoya Morisada, Kazumoto Iijima, Kandai Nozu

**Affiliations:** 1 Department of Pediatrics, Kobe University Graduate School of Medicine, 7-5-1 Kusunoki-cho, Chuo, Kobe, Hyogo 650-0017 Japan; 2 Clinical and Translational Research Center, Kobe University Hospital, Kobe, Japan; 3 Department of Pediatrics, Wakayama Medical University, Wakayama, Japan; 4 Department of Child Health and Welfare (Pediatrics), Graduate School of Medicine, University of the Ryukyus, Nishihara, Okinawa Japan

**Keywords:** Genetics, Medical research, Nephrology

## Abstract

Gitelman syndrome is an autosomal recessive inherited salt-losing tubulopathy. It has a prevalence of around 1 in 40,000 people, and heterozygous carriers are estimated at approximately 1%, although the exact prevalence is unknown. We estimated the predicted prevalence of Gitelman syndrome based on multiple genome databases, HGVD and jMorp for the Japanese population and gnomAD for other ethnicities, and included all 274 pathogenic missense or nonsense variants registered in HGMD Professional. The frequencies of all these alleles were summed to calculate the total variant allele frequency in *SLC12A3*. The carrier frequency and the disease prevalence were assumed to be twice and the square of the total allele frequency, respectively, according to the Hardy–Weinberg principle. In the Japanese population, the total carrier frequencies were 0.0948 (9.5%) and 0.0868 (8.7%) and the calculated prevalence was 0.00225 (2.3 in 1000 people) and 0.00188 (1.9 in 1000 people) in HGVD and jMorp, respectively. Other ethnicities showed a prevalence varying from 0.000012 to 0.00083. These findings indicate that the prevalence of Gitelman syndrome in the Japanese population is higher than expected and that some other ethnicities also have a higher prevalence than has previously been considered.

## Introduction

Gitelman syndrome (GS) is an autosomal recessive inherited salt-losing tubulopathy characterized by metabolic alkalosis, hypokalemia, hypomagnesemia, and hypocalciuria. Patients with GS have clinical manifestations including salt cravings, muscle weakness, cramps, fatigue, dizziness, nocturia, and thirst^1,2^. The gene responsible for GS is *SLC12A3* on chromosome 16q13, which encodes the thiazide-sensitive sodium-chloride cotransporter (NCCT) located on the distal convoluted tubule of the kidney^3^. More than 250 pathogenic variants in the *SLC12A3* gene have been reported to date and biallelic inactivating variants of *SLC12A3* cause GS^2,4,5^.

The prevalence of GS is said to be around 1 in 40,000 people, although it is potentially more common among Asians^6^. This suggests that at least 1% of the population is a heterozygous carrier^7^. However, the exact prevalence of GS is unknown because most cases are thought to be asymptomatic or have nonspecific clinical findings^1^. Therefore, most GS patients have not been diagnosed, because of the absence of a blood examination to check their serum potassium levels. A recent study also reported that patients with GS are more likely to develop type 2 diabetes mellitus^8^. We have previously reported that GS patients tend to have extrarenal symptoms such as short stature, thyroid dysfunction, and epilepsy^4^. QT prolongation is also a significant fatal complication of GS^9^. In addition, heterozygous carriers are expected to be quite common because heterozygosity confers the health benefit of a reduced likelihood of developing hypertension^7^. Taken together, this could suggest that there are far more patients and carriers than expected. However, to date, the actual prevalence of GS has not been examined.

In this study, we performed an examination of the disease prevalence of GS and the number of carriers of GS-causing variants by ethnicity based on information in online genome databases. We have been conducting gene tests for GS in Japan and realized that most cases are asymptomatic and diagnosed by chance during blood tests. However, after diagnosis and receipt of precise information about the clinical symptoms, including salt craving, nocturia, or general fatigue, the patients realize that they have been suffering from symptoms since childhood. We also realized that there are few de novo variants in *SLC12A3* and almost all variants are inherited from the parents^4,5^. Therefore, most of the pathogenic variants that have been reported have been registered in single-nucleotide polymorphism (SNP) databases.

In this study, we included all of the missense or nonsense single-nucleotide variants of the *SLC12A3* gene that have been registered in the Human Gene Mutation Database (HGMD Professional, https://portal.biobase-international.com/hgmd/pro/start.php) as pathogenic variants, and searched for allele frequencies using the two public SNP databases for Japanese populations, the Human Genetic Variation Database (HGVD, http://www.hgvd.genome.med.kyoto-u.ac.jp/) and the Japanese Multi Omics Reference Panel (jMorp, https://jmorp.megabank.tohoku.ac.jp/202001/), as well as the Genome Aggregation Database (gnomAD, https://gnomad.broadinstitute.org/) for other ethnicities. We used the data obtained to calculate and compare the number of patients and carriers of GS in each ethnicity.

In addition, for nine variants with especially high allele frequencies in the Japanese population, which were expected to have a particularly strong impact on the results, we performed several in silico analyses, including Sorting Intolerant From Tolerant (SIFT, https://sift.bii.a-star.edu.sg/), MutationTaster2 (http://www.mutationtaster.org/), Polymorphism Phenotyping v2 (PolyPhen-2, http://genetics.bwh.harvard.edu/pph2/), and Combined Annotation Dependent Depletion (CADD, https://cadd.gs.washington.edu/snv), and segregation analyses using the information from our cohort to determine their pathogenicity. Following those, we confirmed the pathogenicity of most of these variants according to the American College of Medical Genetics and Genomics (ACMG) and the Association for Molecular Pathology (AMP) guidelines (Table 2, Supplementary Tables S2–S10)^10^.

Moreover, a retrospective data analysis study was performed that enrolled outpatients between the ages of 16 and 30 years who had undergone blood tests that included the serum potassium level at least once in the last 10 years at our hospital. We found that many of these patients presented with hypokalemia (defined as less than 3.1 mEq/L) and were suspected to have GS, excluding patients with underlying diseases or on medication that affects the serum potassium level.

## Results

There were 16 and 20 single-nucleotide variants out of the 274 missense or nonsense variants registered in HGVD and jMorp, respectively, among the Japanese population, and 140 in the other ethnicities (Supplementary Table S1).

In the Japanese population, the total allele frequencies of the *SLC12A3* gene variants were 0.0474 in HGVD and 0.0434 in jMorp. Thus, the carrier frequencies were 0.0948 (9.5%) and 0.0868 (8.7%), and the calculated prevalence was 0.00225 (2.3/1000 people) and 0.00188 (1.9/1000 people), respectively. The total allele frequencies of the *SLC12A3* gene variants in the other ethnicities were 0.0117 in non-Finnish Europeans, 0.00433 in Finnish Europeans, 0.0140 in Ashkenazim, 0.00350 in South Asians, 0.0288 in East Asians, 0.00505 in Africans, 0.00602 in Latinos, and 0.0125 in others (Supplementary Table S1). Then, the carrier frequencies for each ethnicity were 0.0234 (2.3%) in non-Finnish Europeans, 0.00865 (0.87%) in Finnish Europeans, 0.0280 (2.8%) in Ashkenazim, 0.00699 (0.70%) in South Asians, 0.0575 (5.8%) in East Asians, 0.0101 (1.0%) in Africans, 0.0120 (1.2%) in Latinos, and 0.0249 (2.5%) in others. The calculated prevalence was 0.000137 (0.14/1000 people) in non-Finnish Europeans, 0.0000187 (0.019/1000 people) in Finnish Europeans, 0.000196 (0.2/1000 people) in Ashkenazim, 0.0000122 (0.012/1000 people) in South Asians, 0.000828 (0.83/1000 people) in East Asians, 0.0000255 (0.026/1000 people) in Africans, 0.0000363 (0.036/1000 people) in Latinos, and 0.000155 (0.16/1000 people) in others (Table 1).

**Table 1:** Frequencies of carriers of *SLC12A3* variants and the prevalence of GS in each ethnicity and database.

Race	Japanese		European non-Finnish	European Finnish	Ashkenazi Jewish	South Asian	East Asian	African	Latino	Other
Data bank	HGVD	jMorp	genomAD							
Carrier	0.0948	0.0868	0.0234	0.00865	0.0280	0.00699	0.0575	0.0101	0.0120	0.0249
Patient	0.00225	0.00188	0.000137	0.0000187	0.000196	0.0000122	0.000828	0.0000255	0.0000363	0.000155

The estimated prevalence of GS per 1000 people in the Japanese population is 2.3 in HGVD and 1.9 in jMorp. The estimated prevalences calculated from the two databases with different regional backgrounds are close together, suggesting that there is little regional bias, and we believe that these data confirm the accuracy of the estimated prevalence in the Japanese population. Regarding the estimated prevalence of GS in other ethnicities, the rate in East Asians was the highest (0.83/1000 people), and it was also higher than previously reported (1/40,000) in European non-Finnish (0.14/1000 people) and Ashkenazi Jewish (0.20/1000 people) populations. It was lower in European Finnish (0.019/1000 people) and South Asian (0.012/1000 people) populations, and it was almost equal in Africans (0.026/1000 people) and Latinos (0.036/1000 people) (Table 1).

For the nine variants which had an allele frequency of 0.001 or higher in the Japanese population, several in silico and segregation analyses of 187 cases from our cohort in a previous report^4^ were performed (Supplementary Tables S2–S10). Based on the results, the pathogenicity of these variants was evaluated according to the ACMG/AMP 2015 guidelines, and only three variants (rs199745548, rs201124663, and rs757776621) were considered to be of “uncertain significance” (Supplementary Table S2). Excluding these three variants, the adjusted total allele frequency in the Japanese population was 0.0422 in HGVD and 0.0406 in jMorp. The adjusted estimated carrier frequency was 0.0844 and 0.0814, respectively, and the adjusted estimated prevalence was 1.78 and 1.65 per 1000 people, respectively (Table 2).

**Table 2:** Adjusted total allele frequencies of *SLC12A3* variants according to ACMG/AMP 2015 guidelines and adjusted carrier frequency and prevalence of GS in a Japanese population.

Race	Japanese	
Data bank	HGVD	jMorp
The adjusted total allele frequency	0.0422	0.0406
The adjusted carrier frequency	0.0844	0.0812
The adjusted prevalence	0.00178	0.00165

The retrospective data analysis study using electronic medical records from Kobe University Hospital had a total of 14,335 subjects, of which 143 patients showed hypokalemia (Supplementary Table S11). The number of cases of hypokalemia was 13, or 0.9 per 1000 people, after excluding those with obvious secondary causes other than GS and those who never showed hypokalemia in subsequent consequential blood tests (Table 3).

**Table 3:** Characteristics and blood test results of patients who showed hypokalemia with no apparent cause other than GS.

No	Age	Sex	Aim of blood test	Symptom	Underlying disease or status	Medication	Blood test											
							Na (mEq/L)	K (mEq/L)	Cl (mEq/L)	Mg (mg/dL)	UN (mg/dL)	Cr (mg/dL)	UA (mg/dL)	Alb (g/dL)	VBG			
															pH	pCO2 (mmHg)	HCO3-(mEq/L	BE (mEq/L)
1	29	F	Screening	Extremity weakness	Pregnancy (GA 28 weeks)	–	139	1.8	96	ND	4.8	0.52	8.1	2.9	ND	ND	ND	ND
2	25	F	Screening	–	Pregnancy (GA 8 weeks), Hashimoto's disease	–	138	2.7	100	ND	7.6	0.51	4.4	4.2	ND	ND	ND	ND
3	22	M	Follow-up	–	Gitelman syndrome	KCl	138	2.8	101	2	12.4	0.82	7.6	4.9	7.405	44.4	27.4	2.4
4	25	F	Follow-up	–	Gitelman syndrome, pregnancy (GA19weeks)	KCl,MgO	135	2.8	100	1.7	6.5	0.4	4.2	3.8	ND	ND	ND	ND
5	30	F	Screening	–	Pregnancy (GA 36 weeks), iron-deficiency anaemia	Sodium ferrous citrate	137	2.8	97	ND	7	0.63	6.3	2.5	ND	ND	ND	ND
6	21	M	Screening	–	Sturge-weber syndrome, Glaucoma	Unknown	134	2.9	102	ND	5	0.28	1.7	4.1	ND	ND	ND	ND
7	26	F	Investigation of Hypokalemia	Headache	Suspected of Gitelman syndrome	KCl, potassium aspartate	138	3	99	1.7	11	0.44	4.7	4.9	7.41	50.4	31.3	5.8
8	16	F	Investigation of proteinuria	–	Oral allergy syndrome	–	140	3.1	101	ND	14.4	0.64	6	4.4	7.364	55	31	4.4
9	18	F	Screening	Joint pain	Dissociative osteochondritis	–	140	3.1	102	ND	12	0.46	4	4.7	ND	ND	ND	ND
10	26	M	Investigation of Hypokalemia	–	Gout	Febuxostat	141	3.1	98	1.9	15.1	0.9	9.2	4.6	7.389	55.1	32.4	6
11	27	F	Investigation of rash	Rash, general fatigue	Urticaria	–	133	3.1	100	ND	9.6	0.82	ND	3	ND	ND	ND	ND
12	29	F	Investigation of abdominal pain	Abdominal pain	Abdominal pain	–	136	3.1	103	1.8	6	0.55	ND	4	ND	ND	ND	ND
13	30	M	Investigation of pharyngitis	Sore throat	Pharyngitis	Ampicillin	140	3.1	105	ND	6.7	0.67	ND	3.5	ND	ND	ND	ND

*GA* gestational age, *ND* no data, *UN* urea nitrogen, *Cr* creatinine, *UA* uric acid, *Alb* albumin, *VBG* venous blood gas, *BE* base excess.

## Discussion

To the best of our knowledge, this is the first study to examine the predicted prevalence and carrier frequency of GS by ethnicity using multiple databases. The calculated prevalence of GS was found to be much higher in East Asians, especially in the Japanese population, at approximately 2.0 in 1000.

In Western countries, although the frequency of heterozygous carriers based on phenotypic expression is estimated to be about 1%, the estimated prevalence from 15 common variants in *SLC12A3* in unrelated subjects from the Framingham Heart Study was 0.48%^7^. Some studies in Chinese populations showed that the allele frequency of heterozygous *SLC12A3* variants was about 3%^6,11^. In a recent study in a Japanese population, the overall total allele frequency of nine variants of the *SLC12A3* gene was 3.2% in 1852 Japanese subjects^12^. However, our current study shows a higher frequency than previous results because we investigated all reported pathogenic variants.

Our retrospective data analysis has shown that the ratio of patients suspected of having GS in our hospital was about 0.9 per 1000 people when hypokalemia was defined by a threshold value of 3.1 mEq/L, referring to the median serum potassium level in GS patients^4^. We put the definition as 3.1 mEq/L because the use of a higher threshold caused the number of patients to increase such that it would be impossible to check all medical records. The prevalence was lower than the estimated prevalence of GS; however, here, the threshold for hypokalemia is set rather low. The number of patients would be expected to increase further if the reference value of hypokalemia was extended to 3.5 mEq/L. Therefore, we consider the results of this retrospective analysis to be in no obvious contradiction with the other results of this paper.

These suggest that numerous patients with GS are not accurately diagnosed because, as mentioned above, most of them are asymptomatic or show nonspecific symptoms. However, in a large survey of quality of life in patients with GS, it was revealed that they suffered from nonspecific symptoms causing a significant reduction in quality of life^1^. Some reports also describe rare extrarenal features, including thyroid dysfunction, epilepsy, and long QT syndrome, which could be fatal^4,13^. Patients with GS are also more likely to develop type 2 diabetes mellitus^8^. There are some reports of end-stage renal failure associated with GS, although a large-scale study based on accurate diagnoses is needed in the future to determine the renal prognosis of GS patients^14^. Based on these findings, it can be assumed that there are many more GS patients than previously reported, and earlier testing, diagnosis, and treatment should be conducted as appropriate. We frequently encounter patients suffering from nonspecific symptoms such as serious general fatigue or frequent nocturia. An accurate diagnosis of GS, the provision of appropriate treatment, and an understanding of the clinical manifestations may enable the symptoms of GS to be improved sooner, and help patients to feel reassured by an understanding of the underlying condition.

Interestingly, it has been reported that carriers of heterozygous variants in the *SLC12A3* gene have a health benefit in that they are less likely to develop hypertension^7^. Hypertension affects one billion people worldwide and is the leading cause of death from stroke, myocardial infarction, end-stage renal disease, and congestive heart failure. Ji et al. reported that heterozygous carriers of the *SLC12A3* gene variants (approximately 1–3% of the general population) showed significantly lower blood pressure and were at a lower risk for cardiovascular events^7^. Alternatively, it should be noted, conflicting reports have been published about blood pressure in carriers^6^. It will be intriguing to investigate whether cardiovascular morbidity and mortality do decrease among heterozygous carriers as the population ages, and further research is warranted.

This study shows that the frequencies of heterozygous carriers of the *SLC12A3* gene variants are about 9% in the Japanese population, 6% in East Asians, and 0.7–2.8% in other ethnic groups, indicating that carrier status is common in most ethnic groups. There are some possible reasons why the carrier frequencies are so high for a disease with an autosomal recessive form of inheritance.

The first reason is that the symptoms in GS are not fatal, do not affect development, and affected individuals are capable of reproduction in almost all cases, so both carriers and patients are able to produce offspring.

Second, geographical factors may have contributed to the high prevalence of GS in the Japanese population. Japan is an island country, and an increased prevalence of a condition may arise from the historical migration of carriers to geographically isolated areas, known as a founder effect. In particular, rs146158333 and rs185927948 are carried by more than 1 in 100 Japanese, while these variants are almost undetectable in other ethnicities. The high rates of these two variants may be due to a founder effect, which could explain the high prevalence of GS in the Japanese.

As for the limitations of this study, the method used for calculating the prevalence of GS was simplistic, as an autosomal recessive form of inheritance was assumed and the possibility of de novo variants was not considered. However, our genetic reports indicate that there are few de novo variants of the *SLC12A3* gene, so their impact is considered to be statistically negligible^4,5^.

In addition, the subjects of this study were limited to previously reported missense and nonsense variants within the exons, so we did not consider the presence of other hotspot variants. Only a heterozygous variant can be detected in 15–20% of patients with GS, which suggests that there could be unknown hotspot variants, such as in intronic regions^15^. However, these limitations are likely to have caused the prevalence of GS to have been underestimated in the current results, which would not affect our conclusion that the prevalence of GS is higher than previously reported. To obtain more accurate data on the prevalence of GS, it is important to further understand the pathogenesis of this disease.

Another limitation of this study is that the pathogenicity determination of the database on which it was based could be incorrect. In the HGMD database, the determination of pathogenicity is defined by computed scoring using a supervised machine learning approach known as Random Forest. It is based upon multiple lines of evidence, including HGMD literature support for pathogenicity and some in silico pathogenicity predictions^16^. However, the genetic variant in rs146158333, for example, is considered pathogenic in the HGMD professional database, and Monkawa et al. reported that three of six patients diagnosed with GS (two of whom were sisters) had this homozygous variant, but also two of 50 healthy individuals had the heterozygous variant^17^. They concluded that additional in vitro studies are required to prove that the variant is responsible for GS.

If the pathogenicity decision for a variant which has an allele frequency of 0.001 or higher is wrong, this can significantly affect the results. However, we have detected such high allele frequency variants in our cohort that are strongly suspected of being pathogenic based on in silico and cosegregation studies. As a result, according to the ACMG/AMP 2015 guidelines, six of the nine variants with the especially high allele frequencies in the Japanese population were considered to be “likely pathogenic” (Supplementary Table S2).

We have also compared the allele frequencies published in the report by Fujimura et al.^4^ and the databases for three variants for which the minor allele frequency (MAF) was more than 0.5% by chi-squared test. As a result, two of the three variants (rs146158333 and rs185927948) showed significantly higher frequencies in the population with GS (Supplementary Table S12). For another variant (rs139329616), although the difference was not significant, it tended to be more common, with an allele frequency of 1.1% in the patient group compared with 0.75% in HGVD and 0.56% in jMorp. These data can provide more evidence for the pathogenicity of these variants. Therefore, although some variants with high allele frequency may include variants of unknown significance, the influence of this point on the result is limited. In this way, the reliability of the database needs to be carefully checked when estimating the prevalence of rare diseases with the research methodology used in this study, and further accumulation of cases is expected.

In conclusion, this study suggests that the prevalence of GS could be higher than previously reported. This is especially true in the Japanese population, in which it affects approximately 2.0 in every 1000 people. Although most GS cases are mild, leaving patients undiagnosed may impair their quality of life, and many such patients could exist. Accurate recognition of the prevalence of GS may help in early testing and diagnosis. The analysis of pathogenic genetic variants in GS is progressing, but some uncertainties remain. As these are overcome, it will become possible to conduct more precise genetic studies and more accurately estimate the number of patients.

## Methods

This work is an epidemiological research study using multiple public databanks. First, we included all single-nucleotide variants in exons of the *SLC12A3* gene (referenced as NM_000339.2) registered in HGMD Professional 2020.1 as pathogenic, then excluded small insertion and deletion variants registered in other SNP databases such as jMorp because there were low numbers of these and their allele frequencies were negligibly low in calculating the prevalence (Supplementary Table S13). Consequently, the number of objective variants was 274. HGMD presents published gene variants for inherited human diseases^16^, and the pathogenicity was evaluated by their scoring system.

Second, we searched the allele frequencies for all 274 variants in each ethnicity using HGVD version 2.3^18^ and jMorp 202001^19^ for the Japanese, and gnomAD ver.2.1.1^20^ for other ethnicities, such as non-Finnish Europeans, Finnish Europeans, Ashkenazim, South Asians, East Asians, Africans, Latinos, and others. HGVD is a database that is managed by Kyoto University and contains information on genetic variants based on exome analysis of 1208 samples and cohort studies of 3248 samples, and it is an analysis of genome sequences of healthy Japanese people. jMorp is a database managed by Tohoku University Tohoku Medical Megabank Organization (ToMMo), and it integrates information on health surveys in the Tohoku region of Japan from cohort studies and analysis of more than 10,000 specimens, including whole genome sequences.

The nine variants which have an allele frequency of 0.001 or higher in the Japanese population (rs199745548, rs146158333, rs201124663, rs759532318, rs757776621, rs79351185, rs139329616, rs200697179, and rs185927948), could have a significant influence on the results of this study. The reason for this definition is that in this study design, the inclusion of variants with allele frequencies of less than 0.001 does not have a significant impact on the estimated carrier frequency or prevalence. To further investigate the pathogenicity of these variants, in silico and segregation analyses were performed. In silico analyses were performed using SIFT, MutationTaster2, PolyPhen-2, and CADD (GRCh37-v1.6). The segregation analysis was based on the cohort published in the report by Fujimura et al.^4^ and included 187 patients, adding two cases (B039-2 and B117) whose genetic diagnosis was confirmed after the publication of the study. We then updated the data for eligible cases (see Supplementary Table S10 for details). Regarding case B094, the father was asymptomatic and had the same variants as the patient, while the mother’s specimen was not obtained. Because the pathogenic variants might be in cis, Sanger sequencing was performed again on the patient’s sample, but no other variants were found in the exon of the *SLC12A3* gene. Since the diagnosis could not be confirmed genetically, this case was excluded from this analysis. Among the cohort, those with at least one of the above nine variants were evaluated along with the results of the genetic tests of as many relatives as could be identified. Based on the results of the in silico and segregation analyses, we evaluated the pathogenicity of the nine variants according to the ACMG/AMP 2015 guidelines^10^. For variants suspected to be pathogenic by more than two in silico analyses, PP3 was added to the criteria. PP1 added the criteria for variants that cosegregated with GS in multiple unrelated families, and PM3 was added for variants that were detected in trans with a pathogenic variant in our cohort (see reference 10 for details of each criteria).

For three variants for which MAF was more than 0.5% (rs146158333, rs139329616, and rs185927948), we compared the allele frequency in Fujimura et al.^4^ (the group of GS patients) and the databases (HGVD and jMorp; the group of general Japanese) by chi-squared test.

Moreover, to confirm the accuracy of the results of the above study, we conducted an additional retrospective data analysis study using electronic medical records. All outpatients between the ages of 16 and 30 years who had blood tests that included the serum potassium levels at Kobe University Hospital between January 2010 and December 2020 were included in the study, and the percentage of patients who showed hypokalemia with no apparent cause other than GS was identified. We set this target age range because GS patients often show symptoms in childhood or young adulthood, and hypokalemia due to other factors may increase at older ages. The definition of hypokalemia in this study was 3.1 mEq/L, referring to the median serum potassium level in GS patients reported by Fujimura et al.^4^. We then checked the medical record for all cases with hypokalemia and omitted cases with underlying diseases or on medication that affects the serum potassium levels, such as eating disorders, periodic quadriplegia, chronic or severe diarrhea or vomiting, drug abuse, medication (such as insulin, diuretics, glucocorticoids, ritodrine hydrochloride, and adrenergic beta-2 receptor agonists), malignant tumors, hyperthyroidism, and hemolysis.

### Calculating the disease prevalence of GS and the number of carriers of GS-causing variants

GS is an autosomal recessive inherited disease that develops because of homozygosity or compound heterozygosity of any pathogenic variant. The frequencies of single-nucleotide variants were summed to determine q, the total minor allele frequency of the *SLC12A3* variant in each ethnicity, and we defined p as the normal allele frequency (p + q = 1). According to the Hardy–Weinberg principle, the carrier frequency is 2pq, but because q is much smaller than 1 as GS is a rare disease, the carrier frequency was determined to be twice the total minor allele frequency (2q), assuming that “p = 1 − q ≒ 1”. Furthermore, the predicted prevalence was calculated as q^2,21^.

Then, we compared the prevalence of GS and the rate of carriers of GS-causing variants in each ethnicity obtained by the above calculation.

### Ethical approval

The study protocol is performed in accordance with the relevant guidelines and with the approval of the Ethics Committee at Kobe University Graduate School of Medicine (IRB approval number: B210007). Since this is a retrospective data analysis study that does not use patient specimens and does not involve any physical invasion to patients, we obtained permission from the committee and substituted individual consent with an opt-out statement on the website of Kobe University Hospital.

## Supplementary Information

Supplementary Information.

## Data Availability

We have provided all data in the text and supplementary data file.
