# Supplementary material for: Examination of the predicted prevalence of Gitelman syndrome by ethnicity based on genome databases
Source: Sci Rep. 2021 Aug 9;11:16099. doi: 10.1038/s41598-021-95521-6 (PMC8352941; doi:10.1038/s41598-021-95521-6)
Supplement: Supplementary file 1 — Supplementary Information. [file 41598_2021_95521_MOESM1_ESM.pdf]

Title:

Examination of the Predicted Prevalence of Gitelman Syndrome by Ethnicity Based on Genome Databases

Authors:

Atsushi Kondo, MD*, China Nagano, MD, PhD, Shinya Ishiko, MD, Takashi Omori, PhD, Yuya Aoto, MD, Rini Rossanti, MD, Nana Sakakibara, MD, Tomoko Horinouchi, MD, PhD, Tomohiko Yamamura, MD, PhD, Sadayuki Nagai, MD, Eri Okada, MD, Yuko Shima, MD, PhD, Koichi Nakanishi, MD, PhD, Takeshi Ninchoji, MD, PhD, Hiroshi Kaito, MD, PhD, Hiroki Takeda, MD, PhD, Hiroaki Nagase, MD, PhD, Naoya Morisada, MD, PhD, Kazumoto Iijima, MD, PhD, Kandai Nozu, MD, PhD

Supplementary Table S1. Allele frequencies of *SLC12A3* gene variants in each ethnicity and database

No	Variant	Amino acid change	rs number	Allele Frequency									
				Japanese		European non-Finnish	European Finnish	Ashkenazi Jewish	South Asian	East Asian	African	Latino	Other
				HGVD	jMorp	genomAD							
1	c.37G>C	Ala13Pro	147200024			0.001091	0	0.0008691	0.0004246	0	0.0002408	0.0001412	0.0006929
2	c.160C>T	Arg54Cys	774753302			0.00002638	0	0	0.00006533	0.00005437	0	0.00002891	0
3	c.179C>T	Thr60Met	371443644	0.0008	0.0002	0.000007742	0	0	0.00003266	0.0007517	0	0.00002822	0
4	c.184G>C	Asp62His	757490496			0.00003871	0.0005178	0	0	0	0	0	0
5	c.202G>A	Glu68Lys	763210286			0.00004646	0	0	0	0	0	0	0
6	c.205C>A	His69Asn	780502516			0.000008794	0	0	0	0	0	0	0
7	c.247C>T	Arg83Trp	201255508		0.0001	0.00004406	0	0	0.00003266	0.00005438	0	0.00002891	0
8	c.322C>T	Arg108Trp	541789117			0.00004671	0	0	0.0005553	0.00005016	0.00004024	0.00002825	0.000139
9	c.363G>C	Glu121Asp	146632606			0.001628	0.00003996	0.0003877	0	0	0.0002818	0.0009333	0.0004171
10	c.391G>A	Glu131Lys	199745548	0.001	0.0002	0.00001788	0.0000469	0	0	0.0001094	0	0	0
11	c.403C>T	Arg135Cys	749742102			0.00006332	0	0	0.0001321	0	0.00006308	0.00005879	0
12	c.404G>A	Arg135His	769047841			0.00002721	0	0	0	0	0.00006325	0	0
13	c.433C>T	Arg145Cys	148945966			0	0	0	0.00003278	0.00005442	0	0.00002896	0.0001631
14	c.434G>A	Arg145His	374324018			0.0002561	0.0001198	0	0.00003279	0	0	0	0.0001385
15	c.457G>A	Val153Met	779074538			0.0001165	0	0	0.00003282	0	0.00004019	0	0
16	c.460A>T	Ile154Phe	748547209			0.00003881	0	0	0	0	0	0	0
17	c.470T>C	Leu157Pro	775047246			0.00001765	0	0	0	0	0	0	0
18	c.473G>A	Arg158Gln	1274973729			0.00001555	0	0	0	0	0.00004026	0	0.0001389
19	c.472C>T	Arg158Trp	1041531529			0.00001554	0	0	0	0	0	0	0
20	c.488C>T	Thr163Met	267607050		0.0001	0.000007813	0	0	0	0.0003524	0	0.00005673	0
21	c.497C>T	Ala166Val	779683214			0.00006483	0	0	0	0	0	0	0
22	c.505G>A	Val169Ile	201650578			0.000045	0	0	0	0.00005507	0	0.0001757	0
23	c.514T>C	Trp172Arg	757792232			0.00001759	0	0	0	0	0	0	0
24	c.533C>T	Ser178Leu	772589653	0.0004	0.0002	0.00001759	0	0	0	0.00005437	0	0.00002891	0
25	c.539C>A	Thr180Lys	146158333	0.0145	0.013	0	0	0	0	0.003458	0	0	0.0001384
26	c.557G>A	Gly186Asp	759426055			0.00001759	0	0	0	0	0	0	0
27	c.575T>C	Ile192Thr	123175433			0	0	0	0	0.00005437	0	0	0
28	c.587G>T	Gly196Val	1290372649			0	0	0	0	0.00005437	0	0	0
29	c.626G>A	Arg209Gln	758035631			0.00003871	0	0	0.00003266	0	0.00004005	0.00002822	0
30	c.626G>C	Arg209Pro	758035631			0.000008792	0	0	0	0	0	0	0
31	c.625C>T	Arg209Trp	28936388			0.00001758	0	0	0.00003244	0	0	0	0
32	c.635G>A	Gly212Asp	1202006117			0	0	0	0	0	0	0	0
33	c.644T>C	Leu215Pro	780594361			0.000008791	0	0	0.00003266	0	0	0	0
34	c.676G>A	Ala226Thr	774753202			0.000008792	0	0	0.00003266	0	0	0	0
35	c.689G>A	Gly230Asp	375990084			0.00007034	0	0	0	0	0	0.00002891	0
36	c.704C>T	Thr235Met	369344478	0.0008	0.0008	0.00004647	0	0	0.00003266	0.0003506	0	0.00005644	0.0004159
37	c.727C>T	Arg243Trp	772187470			0	0.00004759	0	0.00006534	0	0	0.00002892	0
38	c.760G>A	Val254Met	751725614			0.00003098	0	0	0	0.0003007	0	0	0
39	c.775G>A	Asp259Asn	780461639			0.00001758	0	0	0.00003266	0	0.00006152	0	0
40	c.781C>T	Arg261Cys	201124663	0.0021	0.0012	0.0001549	0	0	0	0.0006016	0.00004009	0.00002822	0.0001384
41	c.782G>A	Arg261His	914588619			0	0	0	0	0	0.00006152	0	0
42	c.815T>C	Leu272Pro	568513106			0.00003874	0	0	0	0	0	0	0
43	c.911C>T	Thr304Met	755069436			0.00000879	0	0	0	0.0001087	0	0	0
44	c.910A>C	Thr304Pro	753840283			0.00006153	0	0	0	0	0	0	0
45	c.938C>T	Ala313Val	140551719			0.00003871	0	0	0	0	0.00004005	0	0
46	c.947G>T	Gly316Val	748920885			0.00002323	0	0	0	0	0.0001202	0	0
47	c.961C>T	Arg321Trp	150046661			0.000124	0.0002406	0	0.00003268	0	0	0	0.0002769
48	c.965C>T	Ala322Val	142679083			0.002253	0.0002389	0.006172	0.000294	0.0007517	0.001441	0.001129	0.003461
49	c.1000C>T	Arg334Trp	770702194			0.00003097	0	0	0.00003266	0	0.00004005	0	0
50	c.1046C>T	Pro349Leu	121909383			0	0	0	0	0	0.00006152	0	0
51	c.1049C>T	Ser350Leu	778585043			0.00004396	0	0	0	0	0	0	0
52	c.1067C>T	Ala356Val	1280772093			0.00001548	0	0	0	0	0	0	0
53	c.1077C>G	Asn359Lys	181865675			0	0	0	0	0.00005438	0	0	0
54	c.1079T>C	Ile360Thr	1407947212			0.000008792	0	0	0	0	0	0	0
55	c.1100C>T	Pro367Leu	774478823			0.000008996	0	0	0	0.00005462	0	0	0
56	c.1121G>A	Gly374Glu	773669504			0	0	0	0	0	0	0	0
57	c.1121G>T	Gly374Val	773669504			0.000008925	0	0	0	0	0	0	0
58	c.1124C>A	Thr375Asn	1166885838			0	0	0	0	0	0	0	0
59	c.1132G>A	Ala378Thr	777045692			0	0	0	0	0	0	0.00005803	0
60	c.1145C>T	Thr382Met	187885782			0.0000233	0	0	0	0.0001003	0.00004013	0.0002258	0.0001391
61	c.1175C>T	Thr392Ile	748575829			0.00001766	0	0	0	0.0001088	0	0	0
62	c.1181G>A	Gly394Asp	777815715			0.00000919	0	0	0	0	0	0	0
63	c.1195C>T	Arg399Cys	775931992			0.00000896	0	0	0.00003325	0	0	0.0000293	0
64	c.1196G>C	Arg399Pro	13306668			0.000008947	0	0	0	0	0	0	0
65	c.1216A>C	Asn406His	759532318	0.0004	0.0015	0	0	0	0	0.001144	0	0	0
66	c.1247G>C	Cys416Ser	1429832884			0.00006487	0	0	0	0	0	0	0
67	c.1258G>A	Ala420Thr	137970015			0	0	0	0	0.0002008	0	0.00002825	0
68	c.1261T>C	Cys421Arg	28936387			0.000008835	0	0	0	0	0	0	0
69	c.1262G>A	Cys421Tyr	781342495			0.000008838	0	0	0	0	0	0	0
70	c.1315G>A	Gly439Ser	759377924			0.0002953	0.00007963	0	0	0.00005017	0.00004024	0	0.0002771
71	c.1326C>G	Asn442Lys	775232139			0	0	0	0	0.000641	0	0	0
72	c.1387G>A	Gly463Arg	374163823			0.00003876	0	0	0	0.00005016	0	0.00005646	0
73	c.1388G>A	Gly463Glu	1375515522			0.000008803	0	0	0	0	0	0	0
74	c.1390G>A	Ala464Thr	201945662			0.00003101	0	0	0	0	0.00004011	0	0
75	c.1394C>A	Thr465Asn	745526549			0	0	0	0.00006533	0	0.00006162	0	0
76	c.1424C>G	Ser475Cys	373017321			0.000008684	0	0	0	0	0	0	0
77	c.1432A>G	Lys478Glu	1355705043			0.000008806	0	0	0	0	0	0	0
78	c.1456G>A	Asp486Asn	753523115			0	0	0	0	0.0003427	0	0	0
79	c.1486G>T	Gly496Cys	777612082			0.00001914	0	0	0.00003599	0	0	0	0.0003539
80	c.1519C>T	Arg507Cys	369510226			0.0001565	0	0	0.00003767	0	0	0	0
81	c.1567G>A	Ala523Thr	781137708			0.00002086	0	0	0	0	0	0.00006876	0
82	c.1601A>G	Asn534Ser	780433336			0.000007742	0	0	0	0	0.00004007	0	0
83	c.1606T>C	Phe536Leu	748650798			0	0	0	0	0.0001087	0	0	0
84	c.1625T>C	Leu542Pro	574357286			0.00000879	0	0	0	0	0	0	0
85	c.1644C>G	Phe548Leu	541847708			0.00000879	0	0	0	0	0	0	0
86	c.1664C>T	Ser555Leu	148038173			0.0001006	0.0001196	0	0	0	0.00004005	0	0
87	c.1679C>A	Pro560His	1402444800			0.00001772	0	0	0	0	0	0	0
88	c.1698C>A	Asn566Lys	757776621	0.0021	0.0014	0	0	0	0	0.006766	0	0	0
89	c.1706C>T	Ala569Val	79351185	0.0033	0.0049	0.0001146	0	0	0.000196	0.0004893	0.00006153	0	0.000163
90	c.1709C>T	Ala570Val	201130736			0.0001707	0	0.002702	0.0001307	0.00005012	0.0002404	0	0.0004152
91	c.1712T>C	Leu571Pro				0.000008807	0	0	0	0	0	0	0
92	c.1732G>A	Val578Met	139329616	0.0075	0.0056	0.000007755	0	0	0.00003266	0.00802	0.00004006	0.00005646	0.0008303
93	c.1763C>A	Ala588Glu	121909382			0.00001553	0	0	0	0	0	0	0
94	c.1763C>T	Ala588Val	121909382	0.0004	0.0001	0	0	0	0.00009799	0	0.0001603	0	0
95	c.1837G>A	Gly613Ser	1222807128			0.00006486	0	0	0	0	0	0	0

96	c.1844C>T	Ser615Leu	779160677	0.0004		0.00007684	0	0	0	0.00007579	0.0000562	0	0
97	c.1868T>C	Leu623Pro	121909385	0.0004	0.0009	0	0	0	0	0.00008332	0	0	0
98	c.1919A>G	Asn640Ser	886039754			0	0	0	0	0.00008652	0	0	0.0002231
99	c.1924C>T	Arg642Cys	200697179	0.0017	0.0012	0.00005158	0	0	0.0002189	0.0006117	0.000113	0.0001163	0
100	c.1924C>G	Arg642Gly	200697179			0.00006439	0	0	0	0	0	0	0
101	c.1925G>A	Arg642His	147901432			0	0	0	0	0	0	0	0
102	c.1928C>T	Pro643Leu	140012781			0.00007033	0	0.001738	0.00006533	0	0	0.0002542	0.0006931
103	c.1946C>T	Thr649Met	145337602			0	0	0	0	0.00005014	0.0004012	0.00002824	0
104	c.1963C>T	Arg655Cys	747249619		0.0001	0.00004411	0.00004629	0	0.00003267	0	0	0	0
105	c.1964G>A	Arg655His	121909380			0.0001398	0	0.000193	0.00003267	0	0.0000401	0.00005646	0
106	c.1964G>T	Arg655Leu	121909380			0	0	0	0.00006533	0	0	0	0
107	c.1967C>T	Pro656Leu	140363569			0.0002173	0	0	0	0.0001003	0.0001203	0.00008469	0
108	c.2186G>T	Gly729Val	373901523			0.0001635	0.0001597	0	0.00003266	0.00005016	0	0	0
109	c.2191G>A	Gly731Arg	752101663			0.00004668	0	0	0	0	0.00004021	0	0.0001392
110	c.2204C>G	Pro735Arg	757761069			0.00001761	0	0	0	0	0	0	0
111	c.2221G>A	Gly741Arg	138977195			0.0006833	0	0.0001931	0.00003266	0	0.0001608	0.0001976	0.0002779
112	c.2243C>T	Ser748Leu	771544107			0.0000622	0	0	0.00003266	0.0001505	0	0.00002823	0
113	c.2252C>T	Pro751Leu	368068353			0.00006995	0.0004798	0	0.00003267	0	0.0002009	0	0.000139
114	c.2344G>A	Val782Met	748388143			0.000007743	0	0	0.00003266	0.0002005	0	0	0
115	c.2497T>A	Ser833Thr	146845953			0.0006903	0	0	0	0	0.0002006	0.0004517	0.0002771
116	c.2573T>A	Leu858His	185927948	0.0108	0.0114	0	0	0	0	0.0007612	0	0	0.0001633
117	c.2576T>C	Leu859Pro	121909379			0.0001317	0	0	0	0	0.00004012	0.0004238	0
118	c.2581C>T	Arg861Cys	373899077			0.000155	0	0	0.00003273	0.00005012	0	0.00005648	0
119	c.2582G>A	Arg861His	751929135			0.000008804	0	0	0.00003275	0	0	0	0
120	c.2611C>T	Arg871Cys	754505583			0.00001759	0	0	0	0	0	0	0
121	c.2612G>A	Arg871His	267607051			0	0	0	0.00003268	0.0003508	0	0	0
122	c.2626G>A	Gly876Ser	370301695			0.0001394	0	0	0.00006538	0.0001002	0	0	0
123	c.2642T>C	Met881Thr	752124879			0.00003098	0	0	0	0	0	0	0
124	c.2671C>G	Leu891Val	1386040480			0	0	0	0	0.00005437	0	0	0
125	c.2687G>A	Arg896Gln	369360334			0.00003871	0	0	0.00003266	0	0.00008011	0.00005643	0
126	c.2723T>C	Ile908Thr	1481607874			0	0	0	0	0	0	0.00002891	0
127	c.2827C>T	Arg943Trp	201721269			0.00007913	0	0	0.00009799	0	0	0.0001445	0
128	c.2839C>T	Pro947Ser	772818832			0	0	0	0	0.0003009	0	0	0
129	c.2891G>A	Arg964Gln	202114767	0.0008	0.0001	0.00008514	0.00003981	0.001736	0	0.00005013	0.00004004	0.0001411	0.0005534
130	c.2890C>T	Arg964Trp	559626481			0.00006192	0	0	0	0.00005013	0	0.00005643	0.0001384
131	c.2930G>A	Arg977Gln	186979818			0.00002322	0.001712	0	0	0	0	0.00002822	0.0002768
132	c.2947G>A	Val983Ile	373092349		0.0001	0.00006193	0.0004384	0	0	0.0002506	0	0.00005643	0.0001384
133	c.2965G>A	Gly989Arg	34803727			0.0001088	0	0	0	0	0	0.00008465	0
134	c.2981G>A	Cys994Tyr	199849117			0.0002558	0	0	0	0	0	0.0003668	0.000969
135	c.2996A>G	Tyr999Cys	1358978662			0.00002323	0	0	0	0	0	0	0
136	c.3053G>A	Arg1018Gln	370175770		0.0003	0.00002322	0	0	0	0	0.0001202	0	0
137	c.3052C>T	Arg1018Term	781209989			0	0	0	0.00003266	0	0	0	0.0001629
138	c.3070G>A	Val1024Met	775765261			0.00002637	0	0	0	0	0	0	0
139	c.3077C>T	Thr1026Ile	769248531			0.00001758	0	0	0	0	0	0	0
140	c.3089A>G	Gln1030Arg	762026283			0.00001548	0	0	0	0	0	0	0
		Total		0.0474	0.0434	0.0117	0.00433	0.0140	0.00350	0.0288	0.00505	0.00602	0.0125

Supplementary Table S2. Results of segregation and *in silico* analysis for the variants which have an allele frequency of 0.001 or higher in the Japanese population, and clinical interpretation of genetic variants by the ACMG/AMP 2015 guidelines

No	Variant	Amino acid change	rs number	Allele Frequency		segregation analysis in our cases	In silico Analysis				Clinical Interpretation of genetic variants by ACMG/AMP 2015 guideline (checked criterias)
				Japanese			SHIFT	Mutation Taster	PolyPhen-2	CADD score	
				HGVD	jMorp						
10	c.391G>A	Glu131Lys	199745548	0.001	0.0002	ND	Tolerated(score:0.62)	Polymorphism(prob:1)	Benign(score 0.000)	13.68	Uncertain significance(PM2,BP4)
25	c.539C>A	Thr180Lys	146158333	0.0145	0.013	Co-segregated with GS	Tolerated(score:0.07)	Polymorphism(prob:1)	Benign(score 0.27)	14.19	Likely pathogenic(PM1,PM2,PM3,PP1,BP4)
40	c.781C>T	Arg261Cys	201124663	0.0021	0.0012	ND	Deleterios(score:0.03)	Disease causing(prob:1)	Probably damaging(score 1.000)	25.1	Uncertain significance(PM1,PM2,PP3)
65	c.1216A>C	Asn406His	759532318	0.0004	0.0015	Not determinable	Tolerated(score:0.18)	Disease causing(prob:1)	Probably damaging(score 1.000)	24.5	Likely pathogenic（PM1,PM2,PM3,PP3,PP5）,
88	c.1698C>A	Asn566Lys	757776621	0.0021	0.0014	Not determinable	Tolerated(score:0.08)	Disease causing(prob:1)	Benign(score 0.008)	22.9	Uncertain significance(PM1,PM3,BS1)
89	c.1706C>T	Ala569Val	79351185	0.0033	0.0049	Not determinable	Tolerated(score:1)	Polymorphism(prob:0.939)	Benign(score 0.001)	15.85	Likely pathogenic(PM1,PM2,PM3,PP5,BP4)
92	c.1732G>A	Val578Met	139329616	0.0075	0.0056	Co-segregated with GS	Deleterios(score:0)	Disease causing(prob:1)	Probably damaging(score 1.000)	28.2	Likely pathogenic(PM1,PM2,PM3,PP1,PP3,PP5,BP2)
99	c.1924C>T	Arg642Cys	200697179	0.0017	0.0012	Co-segregated with GS	Deleterios(score:0)	Disease causing(prob:1)	Probably damaging(score 1.000)	33	Likely pathogenic(PM1,PM2,PM3,PM5,PP1,PP3)
116	c.2573T>A	Leu858His	185927948	0.0108	0.0114	Co-segregated with GS	Deleterios(score:0)	Disease causing(prob:1)	Probably damaging(score 1.000)	27.3	Likely pathogenic(PM1,PM2,PM3,PP1,PP3,PP5)

ND: no data

Supplementary Table S3. Segregation analysis for the c.539C>A variant

ID	Patient		Father		Mother	
	Variant1	Variant2	Variant1	Variant2	Variant1	Variant2
A001	c.539C>A(p.Thr180Lys)	c.805_806ins18(p.Val268_Thr269	c.539C>A(p.Thr180Lys)	–	c.805_806ins18(p.Val268_Thr269	–
A006	c.539C>A(p.Thr180Lys)	c.1924C>T(p.Arg642Cys)	c.539C>A(p.Thr180Lys)	–	c.1924C>T(p.Arg642Cys)	–
A009-1	c.539C>A(p.Thr180Lys)	c.2573T>A(p.Leu858His)	c.539C>A(p.Thr181Lys)	–	c.2573T>A(p.Leu858His)	–
A009-2	c.539C>A(p.Thr180Lys)	c.2573T>A(p.Leu858His)	c.539C>A(p.Thr182Lys)	–	c.2573T>A(p.Leu858His)	–
B005	c.539C>A(p.Thr180Lys)	c.539C>A(p.Thr180Lys)	c.539C>A(p.Thr183Lys)	–	c.539C>A(p.Thr180Lys)	–
B015	c.539C>A(p.Thr180Lys)	c.1924C>T(p.Arg642Cys)	c.539C>A(p.Thr184Lys)	–	c.1924C>T(p.Arg642Cys)	–
B038	c.539C>A(p.Thr180Lys)	c.2573T>A(p.Leu858His)	c.539C>A(p.Thr185Lys)	–	c.2573T>A(p.Leu858His)	–
B042	c.539C>A(p.Thr180Lys)	c.539C>A(p.Thr180Lys)	c.539C>A(p.Thr186Lys)	–	c.539C>A(p.Thr180Lys)	–
B053	c.539C>A(p.Thr180Lys)	c.1049C>T(p.Ser350Leu)	ND		c.539C>A(p.Thr180Lys)	–
B056	c.539C>A(p.Thr180Lys)	c.667delT(p.Phe223Serfs*79)	c.539C>A(p.Thr180Lys)	–	c.667delT(p.Phe223Serfs*79)	–
B123	c.539C>A(p.Thr180Lys)	c.2573T>A(p.Leu858His)	c.2573T>A(p.Leu858His)	–	c.539C>A(p.Thr180Lys)	–
B170	c.539C>A(p.Thr180Lys)	c.1868T>C(p.Leu623Pro)	c.539C>A(p.Thr180Lys)	–	c.1868T>C(p.Leu623Pro)	–
B186	c.539C>A(p.Thr180Lys)	c.2573T>A(p.Leu858His)	ND		ND	
B187	c.539C>A(p.Thr180Lys)	c.1868T>C(p.Leu623Pro)	c.539C>A(p.Thr180Lys)	–	c.1868T>C(p.Leu623Pro)	–
B196	c.539C>A(p.Thr180Lys)	c.2891G>A(p.Arg964Gln)	c.2891G>A(p.Arg964Gln)	–	ND	
B227	c.539C>A(p.Thr180Lys)	c.805_806ins18(p.Val268_Thr269	ND		ND	
B232	c.539C>A(p.Thr180Lys)	c.1923C>G(p.Tyr641*)	ND		ND	
N18	c.539C>A(p.Thr180Lys)	c.1732G>A(p.Val578Met)	c.1732G>A(p.Val578Met)	–	c.539C>A(p.Thr180Lys)	–
N23	c.539C>A(p.Thr180Lys)	c.1868T>C(p.Leu623Pro)	ND		ND	
N26	c.539C>A(p.Thr180Lys)	c.539C>A(p.Thr180Lys)	ND		ND	
N27	c.539C>A(p.Thr180Lys)	c.539C>A(p.Thr180Lys)	ND		ND	
N33	c.539C>A(p.Thr180Lys)	c.3053G>A(p.Arg1018Gln)	c.539C>A(p.Thr180Lys)	–	c.3053G>A(p.Arg1018Gln)	–
N42-2	c.539C>A(p.Thr180Lys)	c.1195C>T(p.Arg399Cys)	ND		ND	

Supplementary Table S4. Segregation analysis for the c.1216A>C variant

ID	Patient		Father		Mother	
	Variant1	Variant2	Variant1	Variant2	Variant1	Variant2
B017	c.1216A>C(p.Asn406His)	c.2927C>T(p.Ser976Phe)	c.1216A>C(p.Asn406His)	–	c.2927C>T(p.Ser976Phe)	–

Supplementary Table S5. Segregation analysis for the c.1698C>A variant

ID	Patient		Father		Mother	
	Variant1	Variant2	Variant1	Variant2	Variant1	Variant2
B160	c.539C>T(p.Thr180Lys)	c.1698C>A(p.Asn566Lys)	ND		ND	

Supplementary Table S6. Segregation analysis for the c.1706C>T variant

ID	Patient		Father		Mother	
	Variant1	Variant2	Variant1	Variant2	Variant1	Variant2
B004	c.1706C>T(p.Ala569Val)	c.2927C>T(p.Ser976Phe)	c.1706C>T(p.Ala569Val)	–	c.2927C>T(p.Ser976Phe)	–
N20	c.1706C>T(p.Ala569Val)	c.2573T>A(p.Leu858His)	ND		ND	
N32	c.1045C>T(p.Pro349Ser)	c.1706C>T(p.Ala569Val)	ND		ND	

Supplementary Table S7. Segregation analysis for the c.1732G>A variant

ID	Patient		Father		Mother	
	Variant1	Variant2	Variant1	Variant2	Variant1	Variant2
B075	c.1732G>A(p.Val578Met)	c.2573T>A(p.Leu858His)	c.1732G>A(p.Val578Met)	–	c.2573T>A(p.Leu858His)	–
B124	c.1732G>A(p.Val578Met)	c.2573T>A(p.Leu858His)	ND		ND	
N18	c.539C>A(p.Thr180Lys)	c.1732G>A(p.Val578Met)	c.1732G>A(p.Val578Met)	–	c.539C>A(p.Thr180Lys)	–
N37	c.1732G>A(p.Val578Met)	c.2573T>A(p.Leu858His)	c.2573T>A(p.Leu858His)	–	c.1732G>A(p.Val578Met)	–

Supplementary Table S8. Segregation analysis for the c.1924C>T variant

ID	Patient		Father		Mother		Notes
	Variant1	Variant2	Variant1	Variant2	Variant1	Variant2	
A006	c.539C>A(p.Thr180Lys)	c.1924C>T(p.Arg642Cys)	c.539C>A(p.Thr180Lys)	–	c.1924C>T(p.Arg642Cys)	–	
A010	c.1924C>T(p.Arg642Cys)	c.2029G>A(p.Val677Met)	c.1924C>T(p.Arg642Cys)	–	c.2029G>A(p.Val677Met)	–	
B003	c.1924C>T(p.Arg642Cys)	c.2573T>A(p.Leu858His)	c.1924C>T(p.Arg642Cys)	–	c.2573T>A(p.Leu858His)	–	
B011	c.1924C>T(p.Arg642Cys)	c.2573T>A(p.Leu858His)	ND		ND		
B015	c.539C>A(p.Thr180Lys)	c.1924C>T(p.Arg642Cys)	c.539C>A(p.Thr180Lys)	–	c.1924C>T(p.Arg642Cys)	–	
B018	c.817dupG(p.Ala273Glyfs*38)	c.1924C>T(p.Arg642Cys)	ND		c.818insG(p.Ala273Glyfs*38)	–	
B031	c.1924C>T(p.Arg642Cys)	c.2573T>A(p.Leu858His)	ND		c.1924C>T(p.Arg642Cys)	–	
B043-1	c.1924C>T(p.Arg642Cys)	c.1963C>T(p.Arg655Cys)	ND		c.1963C>T(p.Arg655Cys)	–	siblings
B043-2	c.1924C>T(p.Arg642Cys)	c.1963C>T(p.Arg655Cys)	ND		c.1963C>T(p.Arg655Cys)	–	
B049	c.1924C>T(p.Arg642Cys)	c.1930delC(p.Gln644Serfs*28)	ND		ND		
B077	c.1924C>T(p.Arg642Cys)	c.1926delC(p.Gln644Serfs*28)	c.1926delC(p.Gln644Serfs*28)	–	c.1924C>T(p.Arg642Cys)	–	
B115	c.1924C>T(p.Arg642Cys)	c.2573T>A(p.Leu858His)	c.1924C>T(p.Arg642Cys)	–	c.2573T>A(p.Leu858His)	–	
B138	c.1924C>T(p.Arg642Cys)	c.2573T>A(p.Leu858His)	ND		c.1924C>T(p.Arg642Cys)	–	
B146	c.1924C>T(p.Arg642Cys)	c.3053G>A(p.Arg1018Gln)	ND		c.1924C>T(p.Arg642Cys)	–	
B147	c.1924C>T(p.Arg642Cys)	c.2686C>T(p.Arg896*)	ND		ND		
B157	c.1924C>T(p.Arg642Cys)	c.2573T>A(p.Leu858His)	ND		c.2573T>A(p.Leu858His)	–	
B161	c.1924C>T(p.Arg642Cys)	c.2573T>A(p.Leu858His)	ND		ND		
B165	c.1897dupG(p.E633Gfs56)	c.1924C>T(p.Arg642Cys)	ND		c.1897dupG(p.E633Gfs56)	–	
B175	c.1100C>T(p.Pro367Leu)	c.1924C>T(p.Arg642Cys)	c.1100C>T(p.Pro367Leu)	–	c.1924C>T(p.Arg642Cys)	–	
B203	c.1924C>T(p.Arg642Cys)	c.2573T>A(p.Leu858His)	c.2573T>A(p.Leu858His)	–	c.1924C>T(p.Arg642Cys)	–	
B206	c.1924C>T(p.Arg642Cys)	c.2573T>A(p.Leu858His)	ND		ND		
N01	c.1924C>T(p.Arg642Cys)	c.2573T>A(p.Leu858His)	c.2573T>A(p.Leu858His)	–	c.1924C>T(p.Arg642Cys)	–	
N10	c.139delC(p.His47Thrfs*67)	c.1924C>T(p.Arg642Cys)	ND		ND		
N13-1	c.1195C>T(p.Arg399Cys)	c.1924C>T(p.Arg642Cys)	ND		ND		
N13-2	c.1195C>T(p.Arg399Cys)	c.1924C>T(p.Arg642Cys)	ND		ND		

Supplementary Table S9. Segregation analysis for the c.2573T>A variant

ID	Patient		Father		Mother		Notes
	Variant1	Variant2	Variant1	Variant2	Variant1	Variant2	
A009-1	c.539C>A(p.Thr180Lys)	c.2573T>A(p.Leu858His)	c.539C>A(p.Thr180Lys)	–	c.2573T>A(p.Leu858His)	–	siblings
A009-2	c.539C>A(p.Thr180Lys)	c.2573T>A(p.Leu858His)	c.539C>A(p.Thr180Lys)	–	c.2573T>A(p.Leu858His)	–	
A015	c.2573T>A(p.Leu858His)	c.2927C>T(p.Ser976Phe)	c.2573T>A(p.Leu858His)	–	c.2927C>T(p.Ser976Phe)	–	
A019	c.488C>T(p.Thr163Met)	c.2573T>A(p.Leu858His)	ND		c.488C>T(p.Thr163Met)	–	
B003	c.1924C>T(p.Arg642Cys)	c.2573T>A(p.Leu858His)	c.1924C>T(p.Arg642Cys)	–	c.2573T>A(p.Leu858His)	–	
B011	c.1924C>T(p.Arg642Cys)	c.2573T>A(p.Leu858His)	ND		ND		
B013	c.2573T>A(p.Leu858His)	c.2573T>A(p.Leu858His)	ND		ND		
B025	c.664_666delATT(p.Ile222del)	c.2573T>A(p.Leu858His)	c.664_666delATT(p.Ile222del)	–	c.2573T>A(p.Leu858His)	–	
B031	c.1924C>T(p.Arg642Cys)	c.2573T>A(p.Leu858His)	ND		c.1924C>T(p.Arg642Cys)	–	
B034-1	c.178A>G(p.Thr60Ala)	c.2573T>A(p.Leu858His)	c.178A>G(p.Thr60Ala)	–	c.2573T>A(p.Leu858His)	–	siblings
B034-2	c.178A>G(p.Thr60Ala)	c.2573T>A(p.Leu858His)	c.178A>G(p.Thr60Ala)	–	c.2573T>A(p.Leu858His)	–	
B035-1	c.2537_2538delTT(p.Phe846*)	c.2573T>A(p.Leu858His)	c.2573T>A(p.Leu858His)	–	c.2537_2538delTT(p.Phe846*)	–	siblings
B035-2	c.2537_2538delTT(p.Phe846*)	c.2573T>A(p.Leu858His)	c.2573T>A(p.Leu858His)	–	c.2537_2538delTT(p.Phe846*)	–	
B038	c.539C>A(p.Thr180Lys)	c.2573T>A(p.Leu858His)	c.539C>A(p.Thr180Lys)	–	c.2573T>A(p.Leu858His)	–	
B039-1	c.1925G>A(p.Arg642His)	c.2573T>A(p.Leu858His)	ND		ND		siblings
B039-2	c.1925G>A(p.Arg642His)	c.2573T>A(p.Leu858His)	ND		ND		
B046	c.1930delC(p.Gln644Serfs*28)	c.2573T>A(p.Leu858His)	ND		c.1930delC(p.Gln644Serfs*28)	–	
B058	c.1670-1G>T(IVS13-1G>T)	c.2573T>A(p.Leu858His)	c.2573T>A(p.Leu858His)	–	c.1670-1G>T(IVS13-1G>T)	–	
B067	c.2573T>A(p.Leu858His)	c.3052C>T(p.Arg1018*)	ND		c.3052C>T(p.Arg1018*)	–	
B068	c.2573T>A(p.Leu858His)	c.3052C>T(p.Arg1018*)	c.3052C>T(p.Arg1018*)	–	c.2573T>A(p.Leu858His)	–	
B069	c.2573T>A(p.Leu858His)	c.3053G>A(p.Arg1018Gln)	ND		ND		
B073	c.693_695del(p.Ala232del)	c.2573T>A(p.Leu858His)	c.693_695del(p.Ala232del)	–	ND		
B075	c.1732G>A(p.Val578Met)	c.2573T>A(p.Leu858His)	c.1732G>A(p.Val578Met)	–	c.2573T>A(p.Leu858His)	–	
B081	c.2573T>A(p.Leu858His)	ex7-8 skipping	ND		ex7-8 skipping	–	
B084	c.2573T>A(p.Leu858His)	c.2573T>A(p.Leu858His)	c.2573T>A(p.Leu858His)	–	c.2573T>A(p.Leu858His)	–	
B088	c.488C>T(p.Thr163Met)	c.2573T>A(p.Leu858His)	ND		ND		
B091	c.2573T>A(p.Leu858His)	c.2573T>A(p.Leu858His)	ND		c.2573T>A(p.Leu858His)	–	
B092	c.2573T>A(p.Leu858His)	c.2891G>A(p.Arg964Gln)	ND		ND		
B095	c.1289G>A(p.Cys430Tyr)	c.2573T>A(p.Leu858His)	c.2573T>A(p.Leu858His)	–	c.1289G>A(p.Cys430Tyr)	–	
B098	c.2573T>A(p.Leu858His)	c.2891G>A(p.Arg964Gln)	ND		c.2573T>A(p.Leu858His)	–	
B106	c.247C>T(p.Arg83Trp)	c.2573T>A(p.Leu858His)	c.247C>T(p.Arg83Trp)	–	c.2573T>A(p.Leu858His)	–	
B108	c.2573T>A(p.Leu858His)	c.1195C>T(p.Arg399Cys)	ND		ND		
B109	c.2537_2538delTT(p.Phe846*)	c.2573T>A(p.Leu858His)	ND		ND		
B113	c.2029G>A(p.Val677Met)	c.2573T>A(p.Leu858His)	c.2573T>A(p.Leu858His)	–	c.2029G>A(p.Val677Met)	–	
B115	c.1924C>T(p.Arg642Cys)	c.2573T>A(p.Leu858His)	c.1924C>T(p.Arg642Cys)	–	c.2573T>A(p.Leu858His)	–	
B116	c.2573T>A(p.Leu858His)	c.2686C>T(p.Arg896*)	c.2686C>T(p.Arg896*)	–	c.2573T>A(p.Leu858His)	–	
B117	c.2573T>A(p.Leu858His)	c.3052C>T(p.Arg1018*)	ND		ND		
B123	c.539C>A(p.Thr180Lys)	c.2573T>A(p.Leu858His)	c.2573T>A(p.Leu858His)	–	c.539C>A(p.Thr180Lys)	–	
B124	c.1732G>A(p.Val578Met)	c.2573T>A(p.Leu858His)	ND		ND		
B132	c.911C>T(p.Thr304Met)	c.2573T>A(p.Leu858His)	ND		ND		
B136	c.2573T>A(p.Leu858His)	c.3052C>T(p.Arg1018*)	c.3052C>T(p.Arg1018*)	–	c.2573T>A(p.Leu858His)	–	
B138	c.1924C>T(p.Arg642Cys)	c.2573T>A(p.Leu858His)	ND		c.1924C>T(p.Arg642Cys)	–	
B150	c.1930delC(p.Gln644Serfs*28)	c.2573T>A(p.Leu858His)	c.1930delC(p.Gln644Serfs*28)	–	c.2573T>A(p.Leu858His)	–	
B156	c.1963C>T(p.Arg655Cys)	c.2573T>A(p.Leu858His)	c.1963C>T(p.Arg655Cys)	–	c.2573T>A(p.Leu858His)	–	
B157	c1924C>T(p.Arg642Cys)	c.2573T>A(p.Leu858His)	ND		c.2573T>A(p.Leu858His)		
B161	c1924C>T(p.Arg642Cys)	c.2573T>A(p.Leu858His)	ND		ND		
B163	c.238delC(p.(Arg80Glyfs*34))	c.2573T>A(p.Leu858His)	ND		ND		
B167	c.2573T>A(p.Leu858His)	c.2891G>A(p.Arg964Gln)	ND		ND		
B183	c.179C>T(p.Thr60Met)	c.2573T>A(p.Leu858His)	ND		ND		
B185	c.2573T>A(p.Leu858His)	c.2573T>A(p.Leu858His)	c.2573T>A(p.Leu858His)	–	ND		
B186	c.539C>A(p.Thr180Lys)	c.2573T>A(p.Leu858His)	ND		ND		
B198	c.2573T>A(p.Leu858His)	c.2573T>A(p.Leu858His)	ND		ND		
B201	c.2573T>A(p.Leu858His)	c.3052C>T(p.Arg1018*)	ND		c.3052C>T(p.Arg1018*)	–	
B203	c.1924C>T(p.Arg642Cys)	c.2573T>A(p.Leu858His)	c.2573T>A(p.Leu858His)	–	c.1924C>T(p.Arg642Cys)	–	
B204	c.2573T>A(p.Leu858His)	c.2573T>A(p.Leu858His)	c.2573T>A(p.Leu858His)	–	c.2573T>A(p.Leu858His)	–	
B206	c.1924C>T(p.Arg642Cys)	c.2573T>A(p.Leu858His)	ND		ND		
B209	c.2573T>A(p.Leu858His)	c.2573T>A(p.Leu858His)	c.2573T>A(p.Leu858His)	c.2573T>A(p.Leu858His)	ND		The father is suspected of GS.
B220	c.2573T>A(p.Leu858His)	c.3053G>A(p.Arg1018Gln)	ND		ND		
B223	c.2573T>A(p.Leu859His)	c.2573T>A(p.Leu859His)	ND		ND		
B224	c.1163C>A(p.Arg388Asp)	c.2573T>A(p.Leu858His)	ND		c.2573T>A(p.Leu858His)	–	
B226	c.1163C>A(p.Arg389Asp)	c.2573T>A(p.Leu858His)	ND		c.2573T>A(p.Leu861His)	–	
B229	c.2573T>A(p.Leu860His)	c.2891G>A(p.Arg964Gln)	c.2891G>A(p.Arg964Gln)	–	c.2573T>A(p.Leu860His)	–	siblings
B229-1	c.2573T>A(p.Leu861His)	c.2891G>A(p.Arg964Gln)	c.2891G>A(p.Arg964Gln)	–	c.2573T>A(p.Leu860His)	–	
N01	c1924C>T(p.Arg642Cys)	c.2573T>A(p.Leu858His)	c.2573T>A(p.Leu858His)	–	c1924C>T(p.Arg642Cys)	–	
N02	c.1195C>T(p.Arg399Cys)	c.2573T>A(p.Leu858His)	c.1195C>T(p.Arg399Cys)	–	c.2573T>A(p.Leu858His)	–	
N04	c.2029G>A(p.Val677Met)	c.2573T>A(p.Leu858His)	ND		c.2029G>A(p.Val677Met)	–	
N11	c.2221G>A(p.Gly741Arg)	c.2573T>A(p.Leu858His)	ND		c.2221G>A(p.Gly741Arg)	–	
N12	c.2573T>A(p.Leu858His)	c.2573T>A(p.Leu858His)	c.2573T>A(p.Leu858His)	–	ND		
N19	c.2573T>A(p.Leu858His)	c.2891G>A(p.Arg964Gln)	c.2573T>A(p.Leu858His)	–	c.2891G>A(p.Arg964Gln)	–	
N20	c.1706C>T(p.Ala569Val)	c.2573T>A(p.Leu858His)	ND		ND		
N21	c.139delC(p.His47Thrfs*67)	c.2573T>A(p.Leu858His)	ND		c.2573T>A(p.Leu858His)	–	
N22	c.2573T>A(p.Leu858His)	c.2573T>A(p.Leu858His)	c.2573T>A(p.Leu858His)	–	c.2573T>A(p.Leu858His)	–	
N24-1	c.2573T>A(p.Leu858His)	c.2747+2T>A(IVS23+2T>A)	c.2747+2T>A(IVS23+2T>A)	–	c.2573T>A(p.Leu858His)	–	siblings
N24-2	c.2573T>A(p.Leu858His)	c.2747+2T>A(IVS23+2T>A)	c.2747+2T>A(IVS23+2T>A)	–	c.2573T>A(p.Leu858His)	–	
N31	c.179C>T(p.Thr60Met)	c.2573T>A(p.Leu858His)	ND		ND		
N34	c.960C>G(p.Tyr320*)	c.2573T>A(p.Leu858His)	c.960C>G(p.Tyr320*)	–	c.2573T>A(p.Leu858His)	–	
N36	c.1100C>T(p.Pro367Leu)	c.2573T>A(p.Leu858His)	ND		ND		
N37	c.1732G>A(p.Val578Met)	c.2573T>A(p.Leu858His)	c.2573T>A(p.Leu858His)	–	c.1732G>A(p.Val578Met)	–	
N39	c.2573T>A(p.Leu858His)	c.3052C>T(p.Arg1018*)	c.3052C>T(p.Arg1018*)		c.2573T>A(p.Leu858His)	–	
N40	c.2573T>A(p.Leu858His)	c.2877_2878delAG (p.Arg959Serfs*11)	ND		ND		

Supplementary Table S10. Details of the revised cases

Reference 4			Revision details
ID	Variant1	Variant2	
A001	c.539C>A(p.Thr180Lys)	c.805_806ins18 (p.Thr269delinsAsnTrpArgGlyLeuGlyPro)	For Variant 2, the correct amino acid change was p.Val268_Thr269insIleGlyValValValSerVal, which has been corrected.
B039-1	c.1924C>T(p.Arg642Cys)	c.2573T>A(p.Leu858His)	For Variant 1, the correct variant was c.1925G>A (p.Arg642His), which has been corrected.
B039-2	c.1925G>A(p.Arg642His)	c.2573T>A(p.Leu858His)	Added to this analysis because the genetic diagnosis has been confirmed.
B058	c.1670-1G>T(IVS13G>T)	c.2573T>A(p.Leu858His)	For Variant 1, the correct indication was IVS13-1G>T, which has been corrected.
B073	c.664_666delATT(p.Ile222del)	c.2573T>A(p.Leu858His)	For Variant 1, the correct variant was c.693_695del(p.Ala232del), which has been corrected.
B094	c.539C>A(p.Thr180Lys)	c.2573T>A(p.Leu858His)	Since the genetic diagnosis could not be confirmed, this case was excluded from this analysis.
B108	c.2573T>A(p.Leu858His)	c.3052C>T(p.Arg1018*)	For Variant 2, the correct variant was c.1195C>T(p.Arg399Cys), which has been corrected.
B117	c.2573T>A(p.Leu858His)	c.3052C>T(p.Arg1018*)	Added to this analysis because the genetic diagnosis has been confirmed.
B197	c.539C>A(p.Thr180Lys)	c.2891G>A(p.Arg964Gln)	For ID, the correct ID was B196, which has been corrected.
B203	c.1924C>T(p.Leu642Pro)	c.2573T>A(p.Leu858His)	For Variant 1, the correct amino acid change was p.Arg642Cys, which has been corrected.
B206	c.1924C>T(p.Ley642Pro)	c.2573T>A(p.Leu858His)	For Variant 1, the correct amino acid change was p.Arg642Cys, which has been corrected.
B224	c.1163C>A(p.Arg388Asp)	c.2573A>C(p.Leu858His)	For Variant 2, the correct variant was c.2573T>A, which has been corrected.
B226	c.1163C>A(p.Arg389Asp)	c.2573A>C(p.Leu858His)	For Variant 2, the correct variant was c.2573T>A, which has been corrected.

Supplementary Table S11. Serum potassium levels and characteristics of all patients who presented with hypokalemia

No.	Age	Sex	Serum Pottasium	underlying disease or status	Medication
1	21	F	1.7	eating disorder	
2	29	F	1.8	eating disorder	
3	29	F	1.8	pregnancy	
4	24	M	1.9	Basedow's disease	
5	26	F	2.2	eating disorder	
6	21	F	2.3	eating disorder	
7	30	M	2.3	congenital heart disease	furosemide, spironolactone
8	25	F	2.4	Type1 diabetes mellitus,diabetic nephropathy	insulin
9	27	M	2.4	eating disorder	
10	29	F	2.4	Basedow's disease	
11	30	F	2.4	primary hyperaldosteronism	
12	20	F	2.5	Cushing's disease	
13	28	M	2.5	systemic lupus erythematodes	prednisolone
14	28	M	2.5	suprasellar germinoma, panhypopituitarism	
15	29	F	2.5	peritonsillar abscess, malnutrition	
16	29	F	2.5	eating disorder, drug abuse	
17	26	M	2.6	Type1 diabetes mellitus	insulin
18	26	M	2.6	periodic paralysis	
19	29	F	2.6	imminent abortion	ritodrine hydrochloride
20	16	F	2.7	Type1 diabetes mellitus, hypoglycemic attack	insulin
21	19	F	2.7	Septic shock, Hirschsprung disease,short bowel syndrome	
22	21	M	2.7	periodic paralysis?	
23	22	F	2.7	Sjoegren syndrome, renal tubular acidosis	
24	23	F	2.7	eating disorder	
25	24	F	2.7	drug-induced hyperthyroidism	
26	25	F	2.7	microscopic polyangitis(under hemodialysis)	
27	25	F	2.7	pregnancy, Hashimoto's disease	
28	19	F	2.8	eating disorder	
29	20	M	2.8	eating disorder	
30	21	F	2.8	eating disorder	
31	22	M	2.8	Gitelman syndrome	KCl
32	22	M	2.8	X-linked Alport syndrome, renal tubular acidosis	furosemide
33	24	F	2.8	tuberous sclerosis, ileus, vomiturition	
34	25	F	2.8	Gitelman syndrome, pregnancy	KCl, MgO
35	25	F	2.8	eating disorder	
36	26	F	2.8	eating disorder, systemic lupus erythematodes, schizophrenia	
37	27	F	2.8	IgA nephropathy, malnutrition	
38	28	M	2.8	alcoholic hepatic cirrhosis	
39	29	F	2.8	nephronophthisis, post kidney transplantation, renovascular hyperaldosteronism	
40	30	F	2.8	pregnancy, iron-deficiency anaemia	sodium ferrous citrate
41	30	F	2.8	acute drug intoxication	
42	16	M	2.9	Alport syndrome, end stage kidney disease(under peritoneal dialysis)	
43	19	F	2.9	frequent ralapsing and steroid dependent nephrotic syndrome	prednisolone
44	19	F	2.9	Type2 Bartter syndrome	
45	21	M	2.9	Acute pneumonia	
46	21	M	2.9	Sturge-weber syndrome, Glaucoma	unknown
47	21	M	2.9	Takayasu disease, renovascular hyperaldosteronism	
48	22	F	2.9	osteosarcoma, neuroblastoma post bone marrow transplantation	
49	22	F	2.9	acute drug intoxication	
50	24	M	2.9	acute alcoholism	
51	25	F	2.9	systemic lupus erythematodes, lupus nephritis	furosemide
52	25	F	2.9	eating disorder	
53	26	F	2.9	Crohn disease, malnutrition	
54	27	F	2.9	imminent abortion	ritodrine hydrochloride
55	27	F	2.9	chronic kidney disease, renovascular hyperaldosteronism	
56	27	F	2.9	Sjoegren syndrome, renal tubular acidosis, tubular interstitial nephritis	
57	28	F	2.9	eating disorder	
58	16	F	3	acute drug intoxication	
59	17	M	3	acute drug intoxication	
60	17	F	3	renal arterial stenosis, Fibromuscular dysplasia	
61	18	F	3	argininosuccinicaciduria, hepatic insufficiency	
62	19	F	3	Takayasu disease, hypertensive encephalopathy	
63	19	M	3	germinoma	
64	19	F	3	acute drug intoxication	
65	20	F	3	systemic lupus erythematodes	prednisolone
66	20	F	3	acute drug intoxication	
67	21	F	3	systemic lupus erythematodes	prednisolone
68	21	F	3	autosomal dominant polycystic kidney disease	
69	21	F	3	eating disorder	
70	22	M	3	refractory nephrotic syndrome	
71	22	M	3	severe multiple injury	
72	22	F	3	anaphylaxis, vomiturition	
73	24	F	3	pregnancy, vomiturition	
74	24	M	3	renovascular hypertension, renal tubular acidosis	
75	24	F	3	Behcet's disease, habitual diarrhea	
76	24	F	3	imminent abortion	ritodrine hydrochloride
77	25	M	3	severe multiple injury	
78	25	M	3	chronic granulomatous disease, chronic graft-versus-host disease	
79	25	M	3	Hodgkin's lymphangioma, chronic kidney disease	
80	26	F	3	systemic lupus erythematodes	prednisolone
81	26	F	3	suspected of Gitelman syndrome	KCl,Potassium aspartate
82	28	F	3	anaphylaxis, vomiturition	
83	28	M	3	diffuse large B-cell lymphoma	
84	28	M	3	pituitary gland tumor	
85	29	F	3	eating disorder, hypothyroidism	
86	29	M	3	focal segmental glomerulosclerosis, post kidney transplantation	
87	29	M	3	periodic paralysis	
88	29	F	3	bronchial asthma	Short-acting β2 agonist(Inhalation)
89	16	F	3.1	proteinuria, oral allergy syndrome	
90	16	F	3.1	Diamond-Blackfan anemia, habitual diarrhea	
91	17	M	3.1	Fukuyama congenital muscular dystrophy, malnutrition, skinny	
92	17	F	3.1	acute drug intoxication	
93	17	M	3.1	acute alcoholism, traumatic subarachnoid hemorrhage	
94	17	F	3.1	eating disorder	
95	18	F	3.1	Dissociative osteochondritis	
96	19	M	3.1	LEOPARD syndrome	
97	19	F	3.1	diabetes mellitus, systemic lupus erythematodes, pregnancy(GA15w)	insulin
98	19	F	3.1	Type1 diabetes mellitus	insulin
99	19	F	3.1	Type1 diabetes mellitus, diabetic nephropathy, Basedow's disease	insulin
100	20	F	3.1	osteosarcoma, pertussis	
101	20	F	3.1	Eisenmenger syndrome	furosemide, spironolactone
102	21	F	3.1	acute drug intoxication	
103	21	M	3.1	osteosarcoma	
104	21	F	3.1	mixed connective tissue disease, antiphospholipid syndrome	prednisolone

105	21	F	3.1	systemic lupus erythematodes, akathisia, habitual vomiting	
106	22	F	3.1	frequent ralapsing nephrotic syndrome	prednisolone
107	22	F	3.1	acute drug intoxication, epilepticus	
108	22	F	3.1	eating disorder	
109	23	F	3.1	mixed connective tissue disease	prednisolone
110	23	F	3.1	pregnancy, vomiturition, Behcet's disease	prednisolone
111	23	F	3.1	systemic lupus erythematodes, lupus nephritis, end-stage kidney disease(under peritoneal dialysis)	prednisolone
112	23	M	3.1	severe multiple injury	
113	24	F	3.1	minimal change nephrotic syndrome	
114	24	F	3.1	Campylobacter enteritis, diarrea	
115	24	F	3.1	nephronophthisis	
116	24	M	3.1	frequent relapsing nephrotic syndrome, diarrea	
117	26	F	3.1	pregnancy, vomiturition	
118	26	M	3.1	gout	febuxostat
119	26	F	3.1	myasthenia gravis	prednisolone
120	26	F	3.1	acute drug intoxication	
121	26	F	3.1	aneurysmal bone cyst	
122	27	M	3.1	Fanconi syndrome	
123	27	F	3.1	IgM deficiency	
124	27	F	3.1	urticaria	
125	27	F	3.1	nephrogenic diabetes insipidus	
126	27	M	3.1	chronic viral hepatitis type B,acute-on-chronic liver failure	interfelone
127	28	F	3.1	pregnancy, habitual diarrhea	
128	28	F	3.1	unknown	
129	28	M	3.1	hypothyroidism	
130	28	M	3.1	acute alcoholism, insulin-dependent diabetes mellitus	insulin
131	28	F	3.1	acquired Immunodeficiency syndrome,disseminated nontuberculous mycobacterial infection, malnutrition	
132	28	M	3.1	mandibular bone,malnutrition	
133	28	F	3.1	acute pancreatitis, malnutrition	
134	29	M	3.1	eating disorder, biliary atresia, post liver transplantation, Burugada syndrome	
135	29	F	3.1	eating disorder, epilepsy	
136	29	F	3.1	abdominal pain	
137	29	F	3.1	diabetes mellitus(insulin resistant diabetes?)	insulin
138	29	M	3.1	acute drug intoxication	
139	29	F	3.1	acute hepatic insufficiency, post liver transplantetion	prednisolone
140	29	F	3.1	eating disorder	
141	29	F	3.1	systemic lupus erythematodes, Basedow's disease	prednisolone
142	30	M	3.1	pharyngitis	ampicillin
143	30	F	3.1	systemic lupus erythematodes, lupus nephritis, antiphospholipid syndrome	prednisolone

Supplementary Table S12. Results of the chi-square test for the pathogenic analysis of the three variants with MAF (≥ 0.05) in reference 4 and each database

No	Variant	Amino acid change	rs number	Allele Frequency Japanese		Allele frequency in reference 4	p (chi-square test)	
				HGVD	jMorp		HGVD	jMorp
25	c.539C>A	Thr180Lys	146158333	0.0145	0.013	0.07258	1.42602E-17	1.06375E-21
92	c.1732G>A	Val578Met	139329616	0.0075	0.0056	0.01075	0.48	0.1922
116	c.2573T>A	Leu858His	185927948	0.0108	0.0114	0.2419	4.8446E-213	7.592E-262

Supplementary Table S13. Small deletion and insertion variants on exons in the *SLC12A3* gene among the SNPs registered in jMorp

No	Variant	Amino acid change	rs number	Allele Frequency			
				Japanese	European non-Finnish	East Asian	African
				jMorp	gnomAD		
1	c.547_548dupTC	p.Ile184fs	770513014				0.0002
2	c.790dupG	p.Ala264fs	763856201	0.0002			
3	c.791_792insGTGGTCTCGGTCATTGG	p.Val265fs	753631243	0.0002			
4	c.888dupC	p.Phe297fs		0.0001			
5	c.1366dupC	p.Leu456fs		0.0001			
6	c.1439delT	p.Phe480fs	1369558060				0.0001
7	c.1930delC	p.Gln644fs	779215330	0.0004			
8	c.2089_2095delACCAAGT	p.Thr697fs	1248076886, 771701344		0.0001		
9	c.2877_2878delAG	p.Arg959fs	1323003975, 746623621			0.0026	
10	c.2990_2993dupCGCT	p.Tyr999fs	769798104				0.0001
11	c.723_724insTGCTCCAGGTGAGGCCTG	p.Thr241_Val242insCysSerArgTerGlyLeu		0.0001			
